# The difference between metacognition and mindreading: Evidence from functional near-infrared spectroscopy

**DOI:** 10.3389/fpsyg.2022.1037085

**Published:** 2022-10-26

**Authors:** Zhaolan Li, Wenwu Dai, Ning Jia

**Affiliations:** Department of Education, Hebei Normal University, Shijiazhuang, China

**Keywords:** metacognition, mindreading, functional near-infrared spectroscopy, fusiform gyrus, temporoparietal junction

## Abstract

The relationship between metacognition and mindreading was investigated by examining how well one can monitor their own learning (Self) compared to another person’s learning (Other). Here, we used functional near-infrared spectroscopy (fNIRS) to systematically investigate the brain area activation during metacognition and mindreading. The evidence indicated that metacognition and mindreading are underpinned by distinct neural systems. Metacognition is associated with activation in brain regions important for memory retrieval, such as the fusiform gyrus, while mindreading is associated with activation in brain regions important for understanding and reasoning about others’ intentions, such as the right temporoparietal junction (rTPJ).

## Introduction

In many situations in our daily life, people need to monitor their cognitive processes and those of others. When people monitor their own learning and comprehension of the studied material, it is referred to as metacognition. When they monitor other’s learning and comprehension of the studied material, it is referred to as mindreading (Koriat and Ackerman, 2010; Dai et al., 2017). Although metacognition and mindreading do not monitor the same objects, they both involve meta-representations of the mental world (Suddendorf and Whiten, 2001). In metacognition, one’s own mental states are hierarchically categorized into type I (object level) and type II (meta level) mental states. Meta-level mental states are generated when an individual monitors their own object-level mental states. For example, the belief (meta-level) about how well the material will be learned (object level). Accordingly, the mental states attributed to others in mindreading could also be hierarchically categorized into type II levels concerned with object-level and meta-level performance. In this case, meta-level mental states are generated on the basis of monitoring others’ object-level mental states. It can thus be speculated that the main difference between metacognition and mindreading is at the level of object being monitored. Metacognition generally refers to monitoring one’s own mental states, while mindreading generally refers to monitoring the mental states of others. While the meta-level mental states are actually generated by the observer. Therefore, it is still unclear whether metacognition and mindreading rely on the same underlying processes or two separate mental activities.

To explore the relationship between metacognition and mindreading, two theories have been proposed: *one-system* theory and *two-systems* theory. The *one-system* theory posits that the mechanisms underlying metacognition and mindreading are identical. Namely, if someone’s mindreading ability is impaired, then his or her metacognitive ability is also impaired (Happé, 2003). Studies have indicated that autistic children have difficulty representing mental states in others, compared to healthy children. Nicholson et al. (2021) found autistic children were less accurate than healthy children in metacognitive judgments, suggesting that metacognitive and mindreading abilities may share the same cognitive resources. A study investigating the relationship between metacognition and mindreading utilized a picture book reading task with a sample of preschoolers, and the result indicated a significant association between metacognition and mindreading (Lecce et al., 2015). While *two-systems* theory posits that mindreading and metacognition are two independent capacities, realized in distinct and separable brain networks (Carruthers, 2009). Koriat and Ackerman (2010) conducted a study to explore the differences between metacognition and mindreading. The results showed that in metacognitive condition, metacognitive judgment level for oneself was inversely related to the amount of study time. That is, participants effectively monitored their own learning process, but this trend was not found in the mindreading condition. Elmose and Happé (2014) found that autistic children’s metacognitive monitoring accuracy was higher for non-social memory materials (i.e., building images) than that of social memory materials (i.e., face images). This suggested that the impaired mindreading ability would not affect participants’ metacognitive ability. The research of Tirso and Geraci (2020) showed that participants tend to be more confident in the decisions made by others rather than in their own ability to make decisions. Up to now, at the level of behavioral experiments, there are still controversies between the *one-system* theory and the *two-systems* theory. That means the relationship between metacognition and mindreading cannot be accurately explained through behavioral experiments alone. Therefore, in this study, we aim to investigate the differences in brain region activation between metacognition and mindreading from the perspective of brain mechanism, and further elucidate the relationship between metacognition and mindreading.

In order to further clarify the relationship between metacognition and mindreading, some researchers have studied the neural mechanisms of metacognition and mindreading from the perspective of brain mechanisms. Studies have shown that metacognition is associated with activity in the prefrontal cortex. Fleming et al. (2014) found that patients with lesions to the anterior prefrontal cortex showed a deficit in perceptual metacognitive accuracy when compared to healthy participants. Molenberghs et al. (2016) used functional magnetic resonance imaging (fMRI) to explore the brain areas of metacognition and found that individuals activated the anterior medial prefrontal cortex during metacognition, which suggests that this area of brain is involved in higher-order thinking. Lapate et al. (2020) used the face perception paradigm task to explore the neural basis of metacognition, and found the activation of lateral prefrontal cortex to be associated with metacognitive processes. Metacognition has been shown to engage the prefrontal cortex, as well as the fusiform gyrus. Baird et al. (2013) used fMRI technology to explore the neural mechanism of metacognition, and the results showed that metacognitive ability for memory retrieval predicted greater connectivity between fusiform gyrus and precentral gyrus and other regions. Li et al. (2021) also found that the bilateral fusiform gyrus was related to individual metacognitive activities.

Mindreading mainly activates the dorsolateral prefrontal lobe (dlPFC), the temporoparietal junction (TPJ), and the dorsal anterior cingulate cortex (dACC), etc. (Abu-Akel and Shamay-Tsoory, 2011). The right temporoparietal junction (rTPJ) is the core brain region for mindreading (Hill et al., 2017). Tholen et al. (2020) found that bilateral temporoparietal junction, temporal pole, superior temporal sulcus, precuneus, and anterior medial prefrontal lobe were activated during the mindreading task using fMRI technology. A meta-analysis of fMRI studies found that although there was some overlap in the posterior medial prefrontal lobe and precuneus, the overlap was small (Vaccaro and Fleming, 2018). There is evidence to suggest that metacognition and mindreading brain regions are distinct.

Prior research has utilized fMRI technology to investigate the neural underpinnings of metacognition and mindreading. However, the fMRI experimental environment is relatively closed, which differs significantly from real-life situation and has low ecological validity. Functional near-infrared spectroscopy (fNIRS) is a type of non-invasive brain imaging technology that has been developed in recent years. Compared to fMRI technology, fNIRS has several advantages, including low noise levels and greater tolerance of head movement, which can allow participants to conduct experiments in a more naturalistic environment. It is advantageous to monitor the fluctuations of neural activity while participants are engaged in metacognition and mindreading tasks in a naturalistic setting.

Therefore, according to the research of Hill et al. (2017) and Vaccaro and Fleming (2018), we hypothesize that there is a separation between the neural mechanisms of metacognition and mindreading, specifically as follows: (1) The brain regions activated by metacognition and mindreading are significantly different. (2) Mindreading activated the right temporoparietal junction, which was not engaged during metacognition. To verify the hypothesis, similar experimental paradigms for the metacognition and mindreading tasks were used to minimize the inaccuracies brought on by the disparity in experimental paradigms. And, in order to have better ecological validity than previous fMRI studies, fNIRS was used in our study to explore the brain regions responsible for individual metacognition and mindreading in brain mechanisms.

## Materials and methods

### Participants

Thirty-four undergraduate students (13 men, 21 women; *M*_*age*_ = 21.5, *SD* = 2.48) were randomly recruited from different departments of the university, including education science, mathematics, physics, and literature. All participants were adults of normal intelligence, native Chinese speakers, right-handed, with normal or corrected vision. Participants were excluded if they reported a history of neurological or psychiatric illness, use of psychotropic drugs or substances. At the end of the experiment, each participant was given informed consent and received appropriate financial compensation. This study was conducted following the approval of the local ethics committee (No. 2022LLSC027), and participants were fully informed about the study purpose upon completion.

We conducted power analyses (one-sample two tailed *t*-tests) by using G*Power (Faul et al., 2007), setting α to 0.05, effect size to 0.5, which yielded power = 0.81. It meets the statistical requirements.

### Materials

Sixty Chinese character words were selected from the Frequency Dictionary of Modern Chinese (Beijing Language and Culture University, 1986). Each word has two characters which are nouns. After random combination, 30 Chinese character word pairs were chosen as the formal experimental materials. To ensure the homogeneity of materials in the experiment and eliminate the interference of irrelevant factors on the participants, the word frequency and stroke number of the word pairs used in the experimental materials were statistically analyzed. Word frequency ranged from 0.14 to 3.38, and there was no significant difference between cue word and target word [*M _*cue*_* = 1.13, *M _*target*_* = 1.31, *t*(58) = –0.680, *p* = 0.499]; The number of strokes of word pairs ranges from 7 to 24, and there is no significant difference between the number of strokes of cue words and target words [*M _*cue*_* = 16.13, *M _*target*_* = 15.27, *t*(58) = 0.811, *p* = 0.421]. Each word pair consisted of two Chinese characters words, such as “bao bao—ying er” (官官-婴儿), the words on the left were cue words and the words on the right were target words. All word pairs were divided into six groups.

### Design and procedure

A single variable (conditions: self or other) within-participants block design was used (Pan et al., 2017). In the analysis of behavioral data, the independent variable was the confidence rating of self and other. The primary index of NIR brain imaging analysis is the relative change of oxygenated hemoglobin (Oxy-Hb) concentration (Xu et al., 2019).

The experiment was conducted by using the E-prime 3.0 software (E-Prime 3.0 Psychology Software Tools, Inc., Pittsburgh, PA, United States). The experimental procedure was completed in a shielded room, and all stimuli were presented with the same brightness on a Windows XP computer with a 21-inch monitor. The computer screen had a resolution of 1,920 × 1,080, and the eye-screen distance was approximately 60 cm. All of the word pairs were displayed in black, 40-point Song typeface font on a white background.

The experiment consisted of five phases: a study phase, a metacognition phase, a mindreading phase, a distraction phase, and a cue-recall phase. The order of the metacognition phase and the mindreading phase was balanced among participants.

A study trial started with a fixation cross (duration: 500 ms). Next, a word pair (e.g., 宝宝 -- 婴儿) was presented on the screen, in which the word on the left of the screen was the cue word (宝宝) and the word on the right of the screen was the target word (婴儿). Each word pair was displayed for a duration of 4 s. There were 30 Chinese character word pairs in total, and all word pairs were divided into 6 groups with 5 pairs in each group. After learning one group, the participants rested for 20 s before moving on to the next pair.

A metacognition/mindreading phase also began with the fixation cross (duration: 500 ms). In the self-condition, participants were asked to make predictions about how well they had learned 30 word pairs. A cue word was presented and, then participants were instructed to predict how likely they were to recall the target word in a later test for the maximum time limit of 4 s. The question was as follows: “How likely you are to recall the target word in the subsequent test (0: not at all confident—10: very confident),” This question was first answered in the task by using mouse. After completing the estimation of one set of word pairs, the participants rested for 20 s, and then entered the estimation of the next set of word pairs. All of word pairs are grouped in the same way as in the study stage.

For the other condition, before estimating others, the participants were required to orally report the name of the person they estimated. After that, they pressed the space bar to start this task. The fixation cross (duration: 500 ms) was presented to participants at the start of the task. Then a cue word appeared in the screen. The participants were asked to predict the likelihood that the person they estimated would recall the target word in the subsequent test (0: not at all confident—10: very confident), and each word pair had 4 s to make a judgment. There were six groups of word pairs to estimate, and the word pairs are grouped in the same way as in the study phase. After completing the estimation of one set of word pairs, the participants rested for 20 s, and then entered the estimation of the next set of word pairs.

After completing the previous phase of the task, participants were required to complete a 3-min successive inverse minus 3 task on paper.

A cue recall stage contained all the word pairs that participants had studied. A cue recall phase was started with a fixation cross for 500 ms, then a cue word was presented on the screen. Participants were asked to write down the target word on paper in 8 s. The target items were balanced.

### Functional near-infrared spectroscopy instrument

**FIGURE 1:**
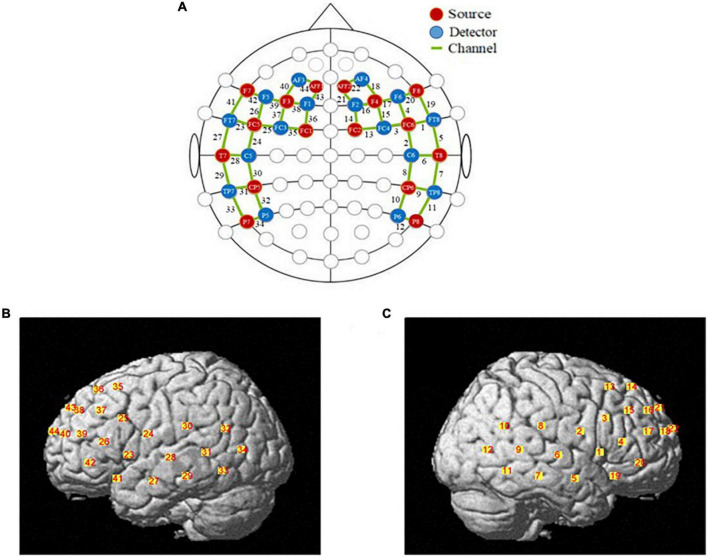
The locations of optodes and channels. **(A)** The locations of optodes and channels with respect to the EEG 10/20 system. The locations of sources (red dots) and detectors (blue dots) and 44 channels (green lines). **(B)** The locations of optical sources and detectors on a standardized 3D head on left view. **(C)** The locations of optical sources and detectors on a standardized 3D head on left view on right view.

The fNIRS data were recorded using a multichannel continuous-wave fNIRS system instrument (NIRx Medical Technologies LLC-NIR-Scout, USA) that consists of sixteen LED light sources and sixteen photodetectors and 44 channels (see Supplementary Table 1). The distance between the detector and source is approximately 3 cm. The detector records relative changes in Oxy-Hb and Deoxy-Hb at a sample rate of 7.81Hz at two wavelengths (760 and 850nm). The location of probe placement and arrangement of specific brain regions was according to previous studies. In this study, we primarily study cognitive functions of the prefrontal cortex region and the temporoparietal junction region. Figure 1 shows the set-up of fNIRS channels. The fNIRS transmitters were specially designed to wrap with a tightly black an elastic nylon cap to ensure that there was no extraneous light interference during the task. The locations of NIRS channels were defined at the central zone of the light path between each adjacent source-detector pair.

Participants were instructed to sit comfortably in a chair and maintain a calm and relaxed position. They were asked to focus on the screen with their minds blank. The visual of the task was presented on a 21-inch thin-film transistor (TFT) screen.

### Data processing

The fNIRS raw data analysis was executed based on SPM with additional modules for a paired samples *t*-test. First, a low-frequency band-pass filter (0.01–0.2 Hz) was applied to eliminate baseline drift, artifacts, and physiological noise. fNIRS records the changes in Oxy-Hb and Deoxy-Hb concentration simultaneously. However, there are some scientific problems with the selection of signals to analyze brain activation. In this research, it mainly focused on the Oxy-Hb signal changes, as it was normally observed to have a higher amplitude than the deoxygenated hemoglobin (Deoxy-Hb) signal. Furthermore, the signal-to-noise (S/N) ratio of Oxy-Hb is better, and the signal is more sensitive to task response. The behavioral performance and Oxy-Hb data were analyzed in Vision 17.0 SPSS using a paired samples *t*-test.

## Results

### Behavioral results

A paired sample *t*-test was conducted on the confidence ratings of self and others. The paired sample *t*-test revealed a significant difference in confidence level between the self and other condition [*t*(33) = –2.298, *p* < 0.05, Cohen’s *d* = 0.20], with higher confidence for others than for themselves (see Supplementary Table 2).

### NIRX results

#### Brain regions activated by metacognition and mindreading

**FIGURE 2:**
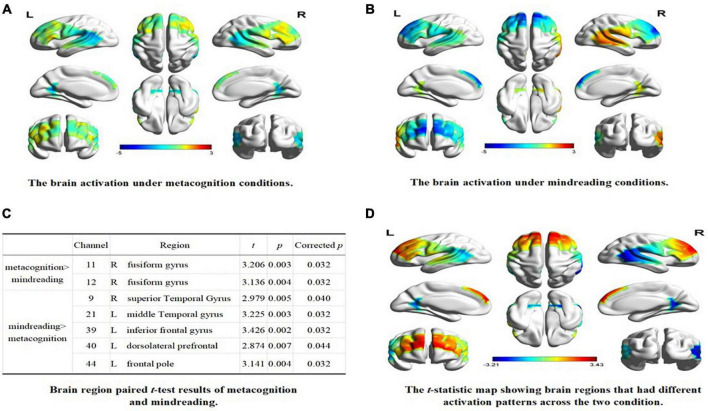
The brain activation under different conditions. **(A)** The brain activation under metacognition conditions. **(B)** The brain activation under mindreading conditions. **(C)** Brain region paired *t*-test results of metacognition and mindreading. **(D)** The *t*-statistic map showing brain regions that had different activation patterns across the two condition.

A one-sample *t*-test was performed on the beta value of each channel under the condition of self-estimation and estimation of others, and the test value was 0. The results showed that, compared with baseline, the channels activated in the metacognition condition were CH2, CH11, CH12, CH32, CH34 (uncorrected, *p* < 0.05), and the corresponding brain regions were inferior central gyrus, right fusiform gyrus, left temporoparietal junction, and left fusiform gyrus (see Figure 2A). CH5, CH7, CH9, CH21, CH36, CH38, CH39, CH40, CH43, CH44 were activated under mindreading conditions (uncorrected, *p* < 0.05). The corresponding brain regions were the right temporoparietal junction, right dorsolateral prefrontal cortex, left prefrontal cortex, left dorsolateral prefrontal cortex, left inferior frontal gyrus, and left frontal pole (see Figure 2B).

The paired sample *t*-test was conducted on the beta values of 44 channels in two types of tasks. After FDR correction, the results showed that: (1) compared with mindreading, CH11 and CH12 were significantly activated during metacognition, corresponding to the right fusiform gyrus; (2) Compared with metacognition, the channels significantly activated during mindreading were CH9, CH21, CH39, CH40, and CH44, corresponding to the right temporoparietal junction, right dorsolateral prefrontal, left inferior frontal gyrus, left dorsolateral prefrontal and left frontal pole (see Figures 2C,D).

## Discussion

In this study, the fNIRS technique was used to investigate the differences between metacognition and mindreading. The results show that the participants tend to give higher confidence levels when estimating others, which is consistent with previous research results (Tirso and Geraci, 2020). The reason for this result is that people lack relevant cues when predicting the internal cognitive state of others (Koriat, 1997), and tend to have a positive view of others. So they have higher confidence in the cognitive ability of others. Tirso and Geraci (2020) also found that, when participants have less access to diagnostic cues and information, they tend to provide inflated judgments of others’ cognitive ability. Moreover, their studies also pointed out that this overconfidence in the cognitive ability of others is consistent in different experimental situations (classroom situations or laboratory) and experimental tasks (in-class exams, grammar tests, logic tests, etc.). This result also proved that there were differences between metacognition and mindreading. The blood oxygen concentration index also showed that there were differences between metacognition and mindreading in brain regions.

The metacognition condition activated the fusiform gyrus, which was consistent with previous findings (Baird et al., 2013). The activation of bilateral fusiform gyrus is associated with the activity of metacognitive monitoring (Li et al., 2021). The activation of bilateral fusiform gyrus was also found in this study, which is consistent with previous research (Baird et al., 2013; Li et al., 2021). It is worth noting that the left temporoparietal junction is also activated during metacognition. Kucyi et al. (2012) showed that there were multiple functional asymmetries in the left and right temporoparietal junction, and the left temporoparietal junction is related to memory retrieval (Reyes et al., 2020). This indicates that metacognitive monitoring will be affected by memory retrieval (Tekin and Roediger, 2021). However, the activation of prefrontal cortex was not detected in this study. This is because the prefrontal regions related to metacognition are mainly medial prefrontal (Lapate et al., 2020), while fNIRS technology can only detect the activity on the surface of the cerebral cortex, and some brain regions highly involved in metacognition (such as medial prefrontal) are partially or completely inaccessible. Therefore, no prefrontal activation was detected in the metacognitive condition.

Under mindreading condition, the right temporoparietal junction, dorsolateral prefrontal cortex, left prefrontal cortex, left inferior frontal gyrus, and left frontal pole were activated, which was consistent with previous findings (Tholen et al., 2020). Mindreading involves speculating about the intentions of others, and the right temporoparietal junction is a brain region specific for mindreading. Activation of this region has been observed during a variety of mindreading tasks. The dorsolateral prefrontal lobe is another brain region involved in predicting other people’s intentions, and activation in this region begins early in neural development. The left inferior frontal gyrus is home to mirror neurons, which have been shown to help individuals understand the actions and intentions of others. Activity in the left frontal pole is often associated with reasoning activity (Urbanski et al., 2016).

The results of the paired sample *t*-test further show a significant separation between metacognition and mindreading. This provides support for the *two-system* theory. Metacognition activates the right fusiform gyrus more than mindreading, which is consistent with previous findings (Vaccaro and Fleming, 2018). According to the Cue-utilization model (Koriat, 1997), participants will try to extract during metacognitive monitoring, and the fusiform gyrus is related to memory retrieval. Compared with metacognition, mindreading activates the right temporoparietal junction, dorsolateral prefrontal cortex, left inferior frontal gyrus, and left frontal pole more, which is consistent with previous findings (Abu-Akel and Shamay-Tsoory, 2011; Tholen et al., 2020). The right temporoparietal junction is the core brain area for mindreading. Hill et al. (2017) used transcranial magnetic stimulation (TMS) and fMRI to find that the functional connectivity of the right temporoparietal junction and the prefrontal cortex is related to the understanding of others’ decision making. The left inferior frontal gyrus is the distribution area of mirror neurons, mirror neural mechanism match the movements of others to their own motor systems, responding to the movements of others with neural circuits of their own movements (Haosheng, 2016), and frontal pole activation related to reasoning activities, indicating that the participants understand others, which is based on his experience to simulate. On the basis of the simulation, the behavior intention of others is speculated.

Our results provide behavioral and neurophysiological evidence for the *two-system* theory. In this study, we sought to investigate differences in metacognition and mindreading by employing a similar experimental design. This allowed us to reduce errors that could have been incurred due to differences in experimental paradigms. At the same time, to have better ecological validity than previous fMRI studies, we used fNIRS to demonstrate the difference between the two from the perspective of brain mechanism.

However, there were some limitations to the present study. Firstly, in this experiment, the participants were required to orally report the name of the person they estimated. But it is still different from the real situation. In future research, two participants can be arranged at the same time, and the participants can be required to make predictions about how well others have learned, after observing the whole learning process of others, so that the research can have higher ecological validity. Secondly, in this study, the researchers did not restrict the range of people that participants estimated. In future studies, the person participants estimated may be grouped according to the social distances between participants and the one they predicted. This would allow researchers to explore whether different social distances would affect the relationship between metacognition and mindreading. Thirdly, the participants of this study are relatively limited in scope. In future research, the participants can be extended to children, the elderly, and special groups to explore how different kinds of participants influence the conclusions of this study. This would help to expand and test the conclusions of this study.

## Conclusion

The present study used fNIRS technology to compare the brain regions activated by metacognition and mindreading. The results showed that metacognition and mindreading were based on two different neural systems, with metacognition primarily engaging the bilateral fusiform gyrus and left temporoparietal junction, among other brain regions associated with memory retrieval. While mindreading ability activated the right temporoparietal junction, left inferior frontal gyrus and left frontal pole, which were related to understanding and reasoning others’ intentions. This finding provides support for the *two-systems* theory from a neural perspective.

## Data availability statement

The raw data supporting the conclusions of this article will be made available by the authors, without undue reservation.

## Ethics statement

The studies involving human participants were reviewed and approved by the Ethical Review Committee of Hebei Normal University. The patients/participants provided their written informed consent to participate in this study.

## Author contributions

All authors listed have made a substantial, direct, and intellectual contribution to the work, and approved it for publication.
