# Supplementary material for: The difference between metacognition and mindreading: Evidence from functional near-infrared spectroscopy
Source: Front Psychol. 2022 Oct 26;13:1037085. doi: 10.3389/fpsyg.2022.1037085 (PMC9643665; doi:10.3389/fpsyg.2022.1037085)
Supplement: Supplementary file 1 [file Data_Sheet_1.docx]

Supplementary Material

# Supplementary Tables

**Supplementary Table 1.** The MNI coordinates and cortical regions of the NIRS channels.

| Channel | Brodmann area | Anatomical label | MNI coordinate | | | percentage of overlap |
| --- | --- | --- | --- | --- | --- | --- |
|  |  |  | x | y | z |  |
| CH1 | 48 | Retrosubicular area | 79 | 18 | 1 | 50.17% |
| CH2 | 43 | Subcentral area | 81 | 4 | 23 | 65.08% |
| CH3 | 44 | pars opercularis | 71 | 23 | 38 | 79.43% |
| CH4 | 45 | pars triangularis Broca's area | 72 | 35 | 15 | 96.07% |
| CH5 | 21 | Middle Temporal gyrus | 82 | 1 | -16 | 100.00% |
| CH6 | 22 | Superior Temporal Gyrus | 85 | -13 | 5 | 66.99% |
| CH7 | 21 | Middle Temporal gyrus | 86 | -30 | -13 | 75.00% |
| CH8 | 2 | Primary Somatosensory Cortex | 85 | -29 | 27 | 50.00% |
| CH9 | 22 | Superior Temporal Gyrus | 84 | -46 | 8 | 67.41% |
| CH10 | 22 | Superior Temporal Gyrus | 78 | -62 | 28 | 56.23% |
| CH11 | 37 | Fusiform gyrus | 80 | -60 | -9 | 84.19% |
| CH12 | 37 | Fusiform gyrus | 73 | -76 | 10 | 79.64% |
| CH13 | 9 | Dorsolateral prefrontal cortex | 50 | 27 | 67 | 79.41% |
| CH14 | 8 | Includes Frontal eye fields | 33 | 45 | 69 | 60.96% |
| CH15 | 45 | pars triangularis Broca's area | 58 | 41 | 45 | 43.64% |
| CH16 | 9 | Dorsolateral prefrontal cortex | 41 | 59 | 48 | 59.91% |
| CH17 | 45 | pars triangularis Broca's area | 59 | 54 | 25 | 59.61% |
| CH18 | 46 | Dorsolateral prefrontal cortex | 44 | 69 | 28 | 73.55% |
| CH19 | 38 | Temporopolar area | 76 | 30 | -18 | 95.68% |
| CH20 | 45 | pars triangularis Broca's area | 68 | 47 | -4 | 51.69% |
| CH21 | 9 | Dorsolateral prefrontal cortex | 27 | 67 | 51 | 90.95% |
| CH22 | 10 | Frontopolar area | 30 | 77 | 30 | 84.56% |
| CH23 | 48 | Retrosubicular area | -79 | 17 | 1 | 58.00% |
| CH24 | 43 | Subcentral area | -81 | 3 | 22 | 69.62% |
| CH25 | 44 | pars opercularis | -72 | 22 | 38 | 78.06% |
| CH26 | 45 | pars triangularis Broca's area | -72 | 35 | 15 | 94.43% |
| CH27 | 21 | Middle Temporal gyrus | -82 | -1 | -18 | 100.00% |
| CH28 | 21 | Middle Temporal gyrus | -85 | -14 | 4 | 50.64% |
| CH29 | 21 | Middle Temporal gyrus | -87 | -31 | -14 | 69.81% |
| CH30 | 2 | Primary Somatosensory Cortex | -85 | -30 | 26 | 44.89% |
| CH31 | 22 | Superior Temporal Gyrus | -84 | -47 | 6 | 52.73% |
| CH32 | 22 | Superior Temporal Gyrus | -77 | -64 | 27 | 76.45% |
| CH33 | 37 | Fusiform gyrus | -79 | -61 | -11 | 92.23% |
| CH34 | 37 | Fusiform gyrus | -73 | -77 | 8 | 74.56% |
| CH35 | 9 | Dorsolateral prefrontal cortex | -51 | 27 | 66 | 79.41% |
| CH36 | 8 | Includes Frontal eye fields | -34 | 44 | 69 | 58.58% |
| CH37 | 45 | pars triangularis Broca's area | -59 | 39 | 46 | 47.72% |
| CH38 | 46 | Dorsolateral prefrontal cortex | -41 | 58 | 49 | 52.04% |
| CH39 | 45 | pars triangularis Broca's area | -59 | 54 | 24 | 62.07% |
| CH40 | 46 | Dorsolateral prefrontal cortex | -45 | 69 | 26 | 81.71% |
| CH41 | 38 | Temporopolar area | -76 | 28 | -19 | 100.00% |
| CH42 | 45 | pars triangularis Broca's area | -68 | 47 | -4 | 54.52% |
| CH43 | 9 | Dorsolateral prefrontal cortex | -27 | 67 | 51 | 83.11% |
| CH44 | 10 | Frontopolar area | -31 | 77 | 30 | 74.71% |

**Supplementary Table 2.** The results of comparing the confidence rating of self and others

| Task type | Confidence | |
| --- | --- | --- |
|  | *M* | *SD* |
| Self | 8.27 | 1.23 |
| Other | 8.52 | 1.23 |
